# COVID-19: On the Disparity in Outcomes Between Military and Civilian Populations

**DOI:** 10.1093/milmed/usab404

**Published:** 2021-10-11

**Authors:** Pete Riley, Michal Ben-Nun, James Turtle, David Bacon, Akeisha N Owens, Steven Riley

**Affiliations:** Predictive Science Inc., San Diego, CA 92121, USA; Predictive Science Inc., San Diego, CA 92121, USA; Predictive Science Inc., San Diego, CA 92121, USA; Leidos Inc., Tysons, VA 22182, USA; Defense Threat Reduction Agency (DTRA) Reachback, Fort Belvoir, VA 22060-6201, USA; Department of Infectious Disease Epidemiology, School of Public Health, Imperial College, London, SW7 2BX, UK

## Abstract

**Introduction:**

The CoronaVirus Disease 2019 (COVID-19) pandemic remains a formidable threat to populations around the world. The U.S. Military, in particular, represents a unique and distinguishable subset of the population, primarily due to the age and gender of active duty personnel. Current investigations have focused on health outcome forecasts for civilian populations, making them of limited value for military planning.

**Materials and Methods:**

We have developed and applied an age-structured susceptible, exposed, infectious, recovered, or dead compartmental model for both civilian and military populations, driven by estimates of the time-dependent reproduction number, *R*(*t*), which can be both fit to available data and also forecast future cases, intensive care unit (ICU) patients, and deaths.

**Results:**

We show that the expected health outcomes for active duty military populations are substantially different than for civilian populations of the same size. Specifically, while the number of cases is not expected to differ dramatically, severity, both in terms of ICU burdens and deaths, is substantially lower.

**Conclusions:**

Our results confirm that the burden placed on military health centers will be substantially lower than that for equivalent-sized civilian populations. More practically, the tool we have developed to investigate this (https://q.predsci.com/covid19/) can be used by military health planners to estimate the resources needed in particular locations based on current estimates of the transmission profiles of COVID-19 within the surrounding civilian population in which the military installation is embedded. As this tool continues to be developed, it can be used to assess the likely impact of different intervention strategies, as well as vaccine policies; both for the current pandemic as well as future ones.

## INTRODUCTION

Since early 2020, CoronaVirus Disease 2019 (COVID-19), caused by severe acute respiratory syndrome coronavirus 2, has been a significant concern for both civilian and military planners alike.^1^ In all likelihood, this will remain the situation for the foreseeable future.^2^ And, while many aspects of the disease are now understood, others remain unknown.^3^

Military populations, as opposed to their civilian counterparts, are unique in a number of important ways.^4^ Specifically, (1) their age demographics are significantly different than most western civilian populations; (2) they are strongly biased toward males; (3) they are generally healthier than the surrounding civilian population; (4) they are likely to follow orders from their superiors; (5) they are generally more separated from other populations; and (6) in general, they are likely to work in closer proximity to one another. Taken together, these factors likely lead to health outcomes from infectious diseases, and COVID-19, in particular, that are different and likely better than the counterpart civilian population in which they are embedded. It is, however, important to quantify to what extent this holds given the potentially severe impacts of both current and future pandemics.

In this study, we test one specific aspect of the disparity between military and civilian populations: age. We hypothesize that differences in the age distributions between these two populations lead to a dramatic reduction in the number of severe cases and deaths for military personnel, which, in turn, significantly reduces the burden placed on military health centers in comparison with similarly sized civilian healthcare facilities. We also discuss how other factors will likely modulate these differences. Finally, we present and describe an online tool^5^ that can be used by military planners to assist them in estimating the likely effects of current or future pandemics.

## MATERIALS AND METHODS

### Data

Age demographics for the U.S. Military were obtained from publicly available online resources.^6^ Here, we do not make any distinctions among the different branches of the U.S. military, recognizing that while some components do have slightly different demographics, these differences are far smaller than the differences between military and civilian populations. Of particular note is that the civilian population is approximately evenly distributed at least from 0 to 69 years of age, whereas the military population is strongly peaked around the 20-29 age group. Given the strong dependence of COVID-19 outcomes with age,^7^ this comparison suggests that military personnel will, in general, suffer less severity and mortality than the general population.

Data used to drive the model, including probabilistic estimates for individuals moving from one compartment to another, were obtained from several sources.^8,9^

### Models

**FIGURE 1.**
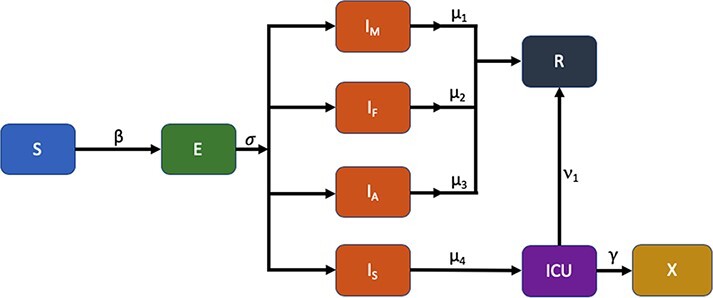
Susceptible, exposed, infectious, and recovered model used to estimate the total number of cases (incidence), the number of intensive care unit (ICU) cases, and the number of deaths.

We developed an age-structured metapopulation model that includes the following compartments: susceptible, exposed, infectious, recovered, or dead. The logic for the model is summarized in Figure 1, which also includes the transmission coefficients for stochastically moving an individual from one compartment to another. The details have been described in more detail elsewhere,^10^ and the underlying code used to generate the results is available in a GitHub repository.^11^ Here, we limit ourselves to a few brief remarks. First, for this study, we developed a simple model for modulating the basic reproduction number, *R*(*t*), as a function of time:

$$\begin{equation*}R(t)=\left\{\begin{array}{ll}R_{1}&t\leq t_{1}\\R_{2}&t \gt t_{1}\end{array}\right.\end{equation*}$$

The value of *R*(*t*) was smoothly changed between the two values, and we included the limiting case that *R*_1_ = *R*_2_ = constant. In particular, for this study, we illustrate three cases: (1) *R*_1_ = 2.5; (2) *R*_1_ = 1.25; and (3) *R*_1_ = 2.5/R_2_ = 0.9. In general, using the web-based app,^5^ the user has more freedom to choose how *R*(*t*) varies with time and/or whether to use *R*(*t*) that has been determined by fits to the available data.

## RESULTS

To illustrate the disparity between military and civilian COVID-19 outcomes, we consider a hypothetical population of 40,000 people, which would correspond to a large military installation or a small U.S. town. For example, Fort Hood military base, located 100 km north of Austin, has an active-duty population of more than 35,000 personnel, in addition to 68,000 family members, more than 103,000 retirees, as well as 40,000 other personnel, such as army reserve, contractors, and civilian employees. Similarly, Coppell City, also in Texas, has a population of 38,700.

**FIGURE 2.**
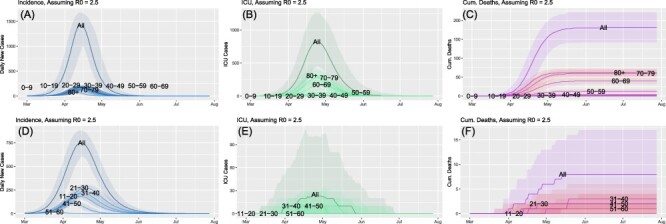
Comparison of incidence and outcomes between civilian (top row) and military (bottom row) populations for constant *R*_0_ = 2.5. Incidence (A, D), number of intensive care unit (ICU) cases (B, E), and the cumulative number of deaths (C, F).

Figure 2 compares incidence (A and D), intensive care unit (ICU) cases (B and E), and cumulative deaths (C and F) for the civilian (top row) and military (bottom row) panels for an idealized scenario where *R*(*t*) remains at 2.5 throughout the first wave. Although this is certainly higher than most U.S. populations maintained throughout the majority of 2020, it is representative of the initial estimates^12^ and would correspond to unmitigated spread, but serves to illustrate our points more clearly by forecasting a rapid outbreak that sweeps through the population relatively quickly (March through May). Several points are worth noting. First, civilian incidence peaked in mid-April with a value of ∼1,400 cases. Second, almost 2 weeks later, ICU cases peaked at a value of just over 800. It is worth noting that this is ICU incidence, not prevalence, which would show a modified profile. Third, cumulative deaths increased exponentially until mid-April, reaching an inflexion point, and then increasing evermore slowly, peaking at a value of ∼180. Based on a population size of 40,000 people, these results suggest a mortality rate of 0.45% (which is distinct from the observed case fatality rate [CFR]). It is also worth noting that the cumulative number of cases was 34,724, resulting in an infection fatality ratio of 0.52%. Fourth, both severity and deaths (the separate curves under the main curve in each panel) showed a strong dependency on age. More intriguing, however, is the temporal phasing of the peaks for each age group. For example, the oldest age group (80+) peaked earlier in time in terms of the number of cases, the number of ICU cases, and the number of deaths. Fifth, despite the oldest age group having the smallest representation in the total population, they made the dominant contribution to ICU cases and, hence, deaths. This also provides an explanation for why the overall peak in ICU cases preceded the peak in incidence since each curve is driven by the opposite extreme in age.

Panels D through F of Figure 2 show analogous results for the hypothetical military installation with the same-size population. The peak number of cases occurred slightly later and is almost half the size of the civilian population. The timing of the overall peak in cases is now driven by the 20-29 age group, with contributions from the two age groups surrounding it providing the next largest contributions. As with the civilian age–group curves, there is a net shift to earlier times with increasing age. However, for the military population, the oldest age group, which is the most sparsely populated, makes only a very modest contribution to the total number of cases (although they peaked more than 2 weeks earlier than the overall peak). Again, the drift to earlier times for the peaks in cases translates to the counterintuitive result that ICU cases peaked slightly before the total incidence profile. However, it is equally important that the size of the ICU peak, at ∼26 cases, was considerably smaller than the civilian peak. While incidence was reduced by almost a factor of 2 between civilian and military populations, ICU cases were reduced by a factor of 30. Of course, given the small total number of cases, these should be viewed as illustrative numbers, rather than definitive. Finally, military deaths were reduced to eight, representing a factor of 22.5 reduction over civilian values. Also of note, however, is that the eventual asymptotic rollover of the cumulative deaths continued slightly longer for the military population; that is, reaching the state of zero deaths took slightly longer for the military, a point that can also be seen from the daily ICU curves, which did not reach zero until late May.

**FIGURE 3.**
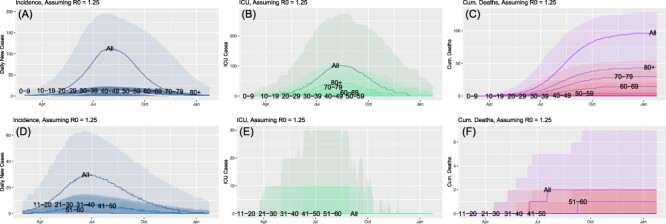
Comparison of incidence and outcomes between civilian (top row) and military (bottom row) populations for constant *R*_0_ = 1.25. Incidence (A, D), number of intensive care unit (ICU) cases (B, E), and the cumulative number of deaths (C, F).

The preceding scenario assumed a constant reproduction number, *R*_0_, of 2.5, which, while reasonable during the initial phases of the outbreak among many civilian populations,^1^ was not sustained in many developed countries.^12^ In fact, many regions were able to reduce *R*(*t*) to values much closer to one.^12^ To address this, in Figure 3, we explore the differences between military and civilian populations where *R*(*t*) takes a constant value of 1.25. Focusing first on the civilian population (A-C), we note that the overall profile has broadened considerably now, lasting from March 2020 through January 2021, as opposed to March through June 2020. The peak incidence has also reduced by approximately an order of magnitude; however, because of the extended duration of the outbreak, the area under the curve is not comparably lower. This is reflected in the total number of deaths, which decreased from 180 to approximately 90; decrease in a factor of 2. It is also of note that the timing of the peak shifted from mid-April to late July, a delay of approximately 10 weeks.

Comparing these civilian results with those from a comparably sized military population (FiG. 3[D-F]) again illustrates similar disparities in relation to the civilian population as discussed above. For example, the peak in incidence for the military population shifts earlier by approximately a month. Additionally, the military population suffers only two total deaths, compared to 90 in the civilian population. Given the small number of deaths, it is not possible to reliably make any quantitative statistical inference from these results. At best, we can state that, again, for this scenario, mortality was substantially lower for the military population.

**FIGURE 4.**
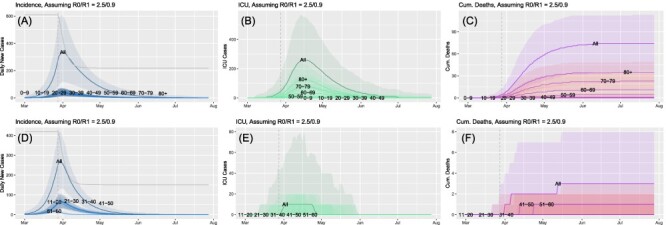
Comparison of incidence and outcomes between civilian (top row) and military (bottom row) populations for a time-varying *R*(*t*), initially set to 2.5 and then dropping to 0.9 at the point indicated by the vertical dashed line in each panel. Incidence (A, D), number of intensive care unit (ICU) cases (B, E), and the cumulative number of deaths (C, F).

Finally, and more realistically, we consider the case where *R*(*t*) was initially set to 2.5, but then smoothly dropped to 0.9 (as indicated by the vertical dashed line), which reflects a more realistic (although optimistic) change in behavior (Fig. 4). The initially large transmission rate led to substantially more cases, particularly during the peak of the wave in the civilian population (∼325, panels A through C). In fact, this peak would have been substantially larger had interventions not been implemented in late March, which drove the evolution along a much shallower path. The total number of ICU cases for the civilian population exceeded 350, and this resulted in 74 deaths. For the counterpart military population, the total number of cases peaked at ∼270; however, only 10 of them were admitted to the ICU. Of these, three patients died. Again, the disparity in both ICU and death between the civilian and military populations is striking, with the latter suffering only 4% (3/74) of deaths in comparison with the civilian population. Similarly, at the peak, military centers would likely only need 3.7% (10/270) of the ICU resources required for the civilian population.

## DISCUSSION

Our results are not surprising. Intuitively, one would expect that a population composed of younger and arguably healthier individuals would not suffer the same impact as a comparably sized civilian population. On the other hand, we believe it is remarkable that the disparity is so substantial. The predicted number of severe cases, i.e., requiring treatment in the ICU, is significantly smaller. Specifically, in terms of total ICU days, we estimate this to be 1,076 for the military population, but 25,324 for the civilian population; a difference of a factor of 24. Given reasonable estimates for the number of ICU beds available to active duty military personnel, saturation is extremely unlikely.

Our analysis considered only active duty personnel. In most military installations, however, they live and interact with their families as well as the broader, surrounding civilian population. Thus, while the underlying transmission dynamics of the disease will be driven by this larger population, it remains the case that the active duty sub-population will suffer better health outcomes calculated here.

Our results highlighted a counterintuitive phenomenon—that the peak incidence for the oldest populations in both the civilian and military populations precedes the overall peak in incidence, which is controlled primarily by the aggregated effects of the younger population. This has an important consequence for military projections in that the peak in ICU demand will likely occur at, or earlier than, the observed peak in incidence, leading to the unfortunate consequence that it may not be possible to anticipate peak demand for ICU capabilities based on observing the peak in incidence. Or, stated another way, when declines in incidence are observed, declines in ICU demand will likely have already started. However, it is also important to emphasize that prevalence within the population, not incidence, is what is reported. Moreover, it is worth pointing out that our model was necessarily idealized in prescribing the same *R*(*t*) profiles for all age groups. In reality, it is likely that those in the older categories shielded themselves substantially more than those in the younger groups, which would have displaced the peak later in time.

Although not explicitly explored here, we note several other fundamental epidemiological differences between military and civilian populations, which could potentially limit the applicability of these results. First, the former is more likely to obey intervention orders. As such, it is likely that military personnel would wear masks and maintain stricter social distancing behavior. In turn, this would drive down *R*(*t*) to values at, or below, one more quickly. Second, military personnel are overwhelmingly male, which suggests a modest but not insignificant increase in ICU admission and death^13^ versus a civilian population composed of approximately equal numbers of males and females. Third, military personnel, even when age-adjusted, are likely healthier than their equivalent civilian counterparts, given the requirements for admission as well as ongoing exercise programs. Fourth, as vaccines are distributed, there will likely be significant differences in rates between civilian and military populations. Although age demographics remain the dominant effect driving differences in outcomes between military and civilian populations, these additional factors undoubtedly play minor, but not insignificant roles. In future studies, we plan to incorporate them into our analysis.

Finally, we note that the results presented here can be recreated along with a number of other types of exploratory and/or planning scenarios using the online tool, DRAFT.^11^

## CONCLUSIONS

In this report, we have used a parsimonious model for investigating the transmission and severity of COVID-19 under several case scenarios for both military and civilian populations. Our results support our hypothesis, that differences in the age distributions between military and civilian populations dramatically reduces the number of severe cases and deaths in the former, which, in turn, suggests that the burden placed on military health centers will be significantly less than civilian healthcare facilities supporting comparably sized populations. As part of this investigation, we developed a tool that can be used by military planners to estimate the impact of COVID-19 on their installation, which we anticipate can help support decisions over the next year or more.
